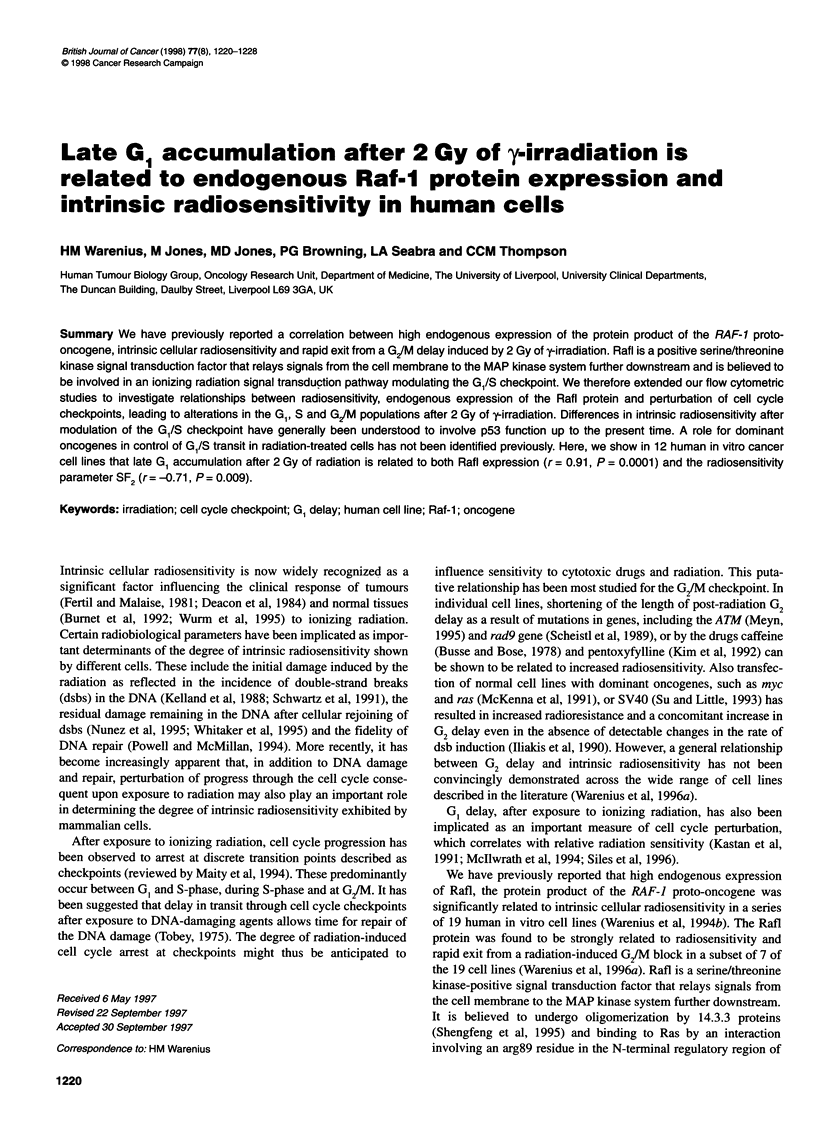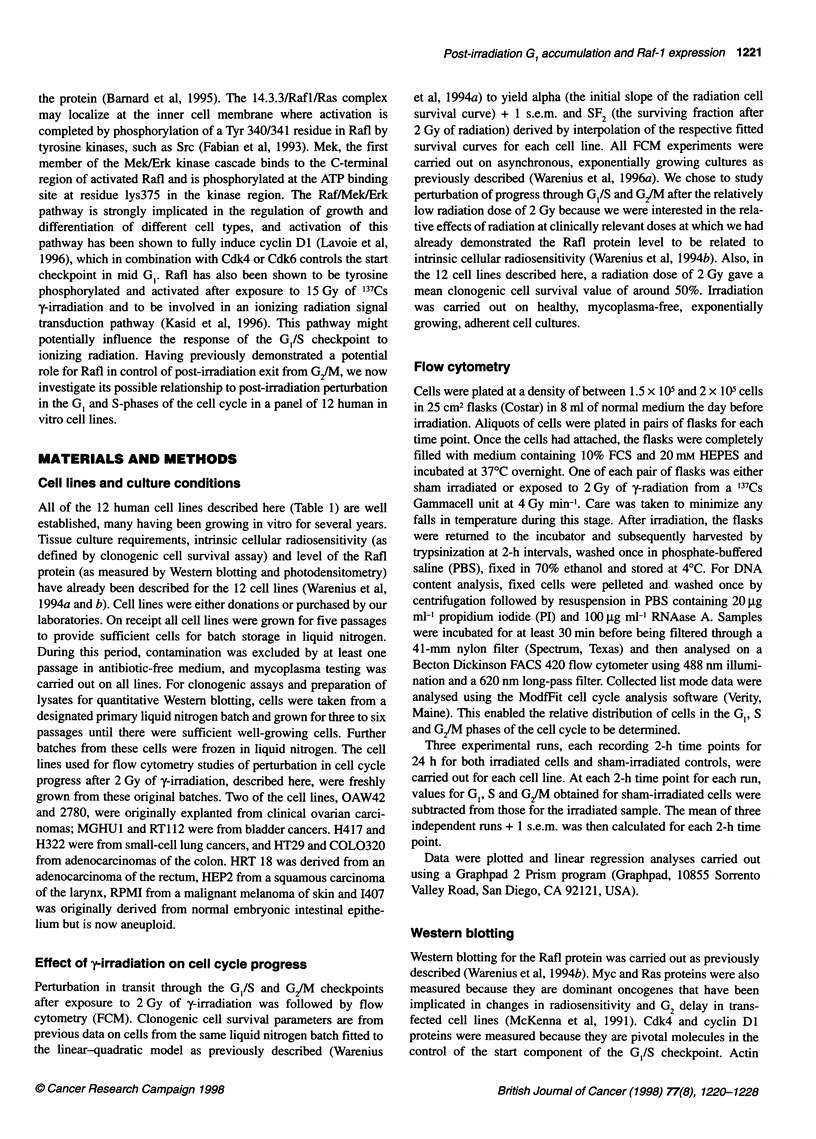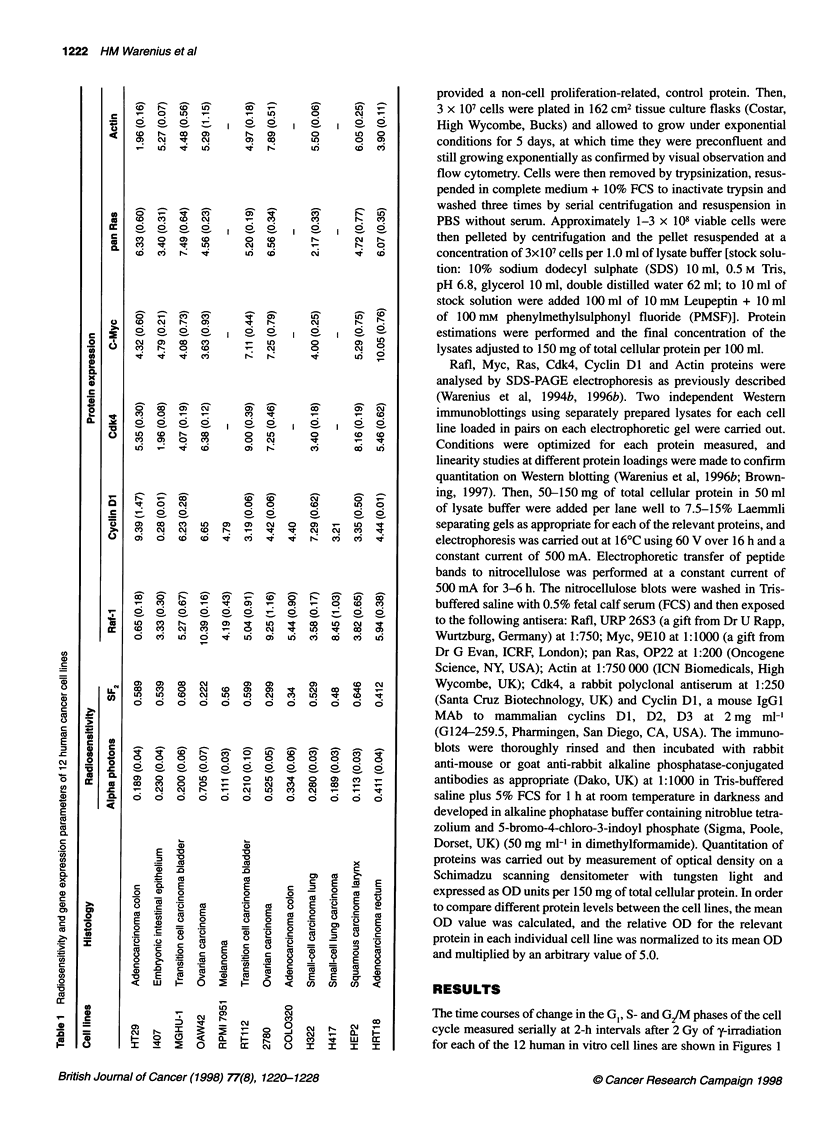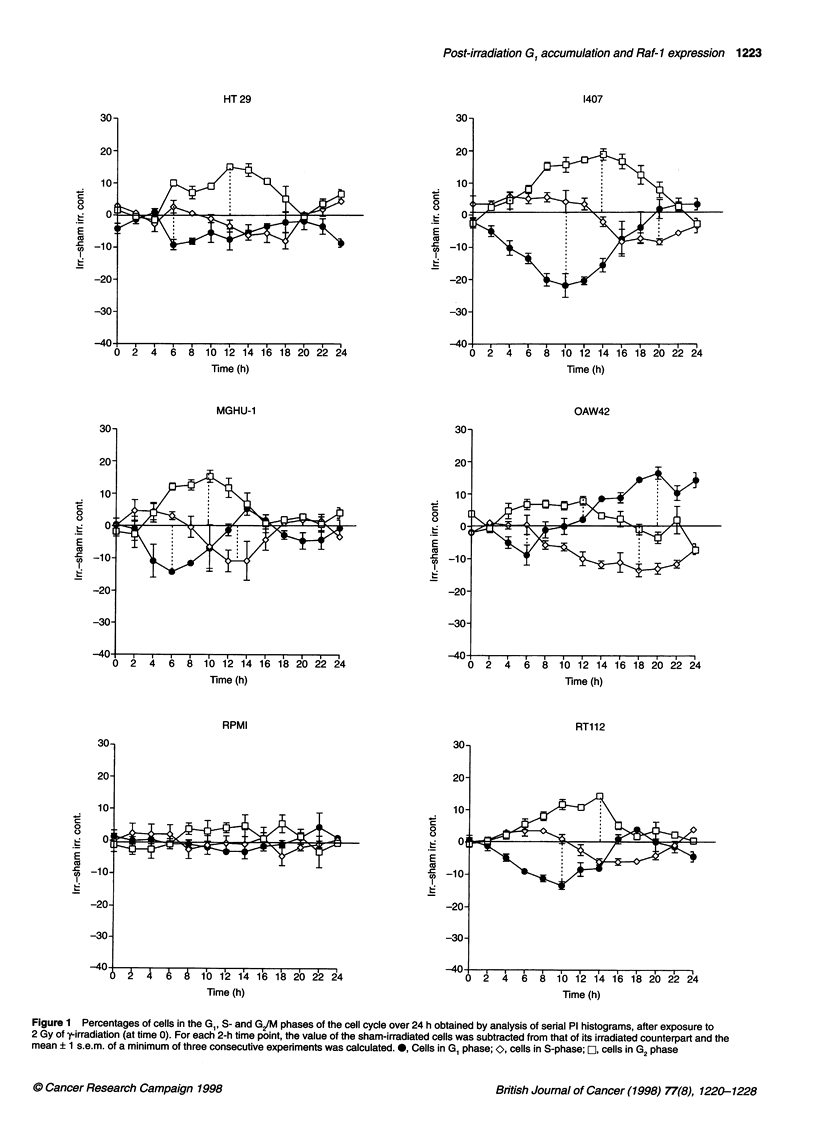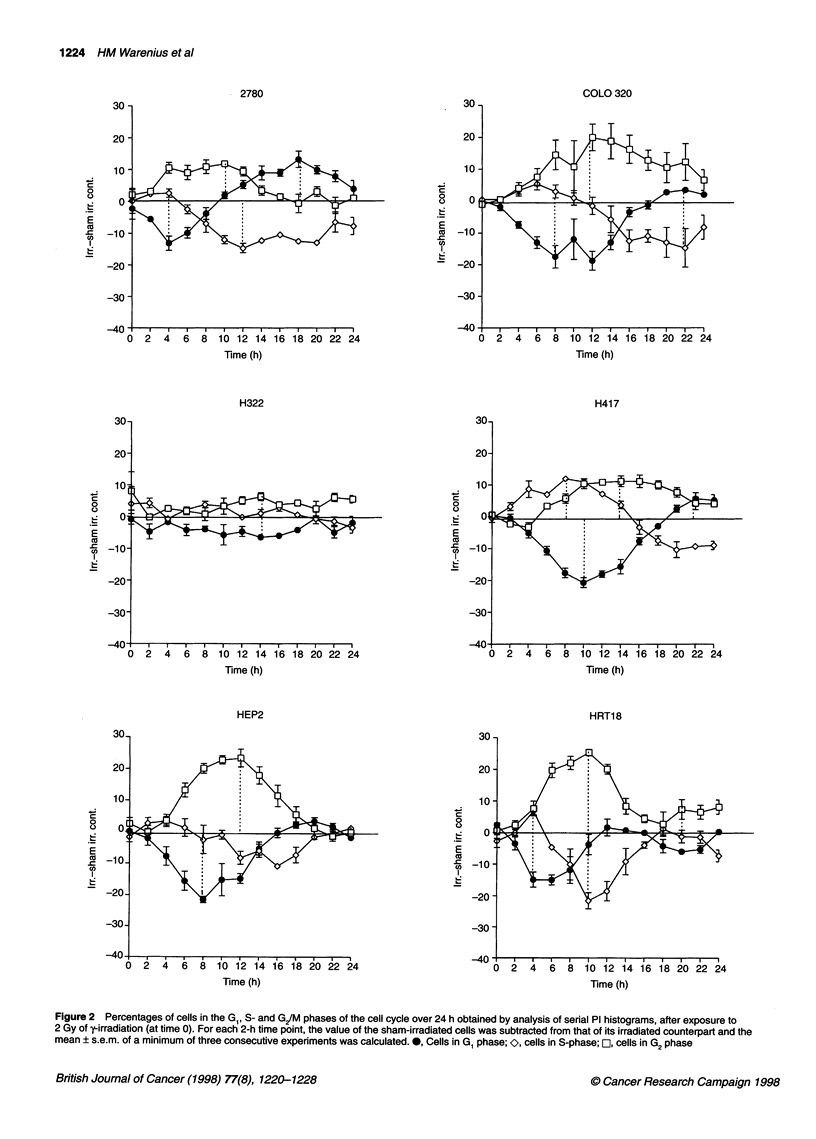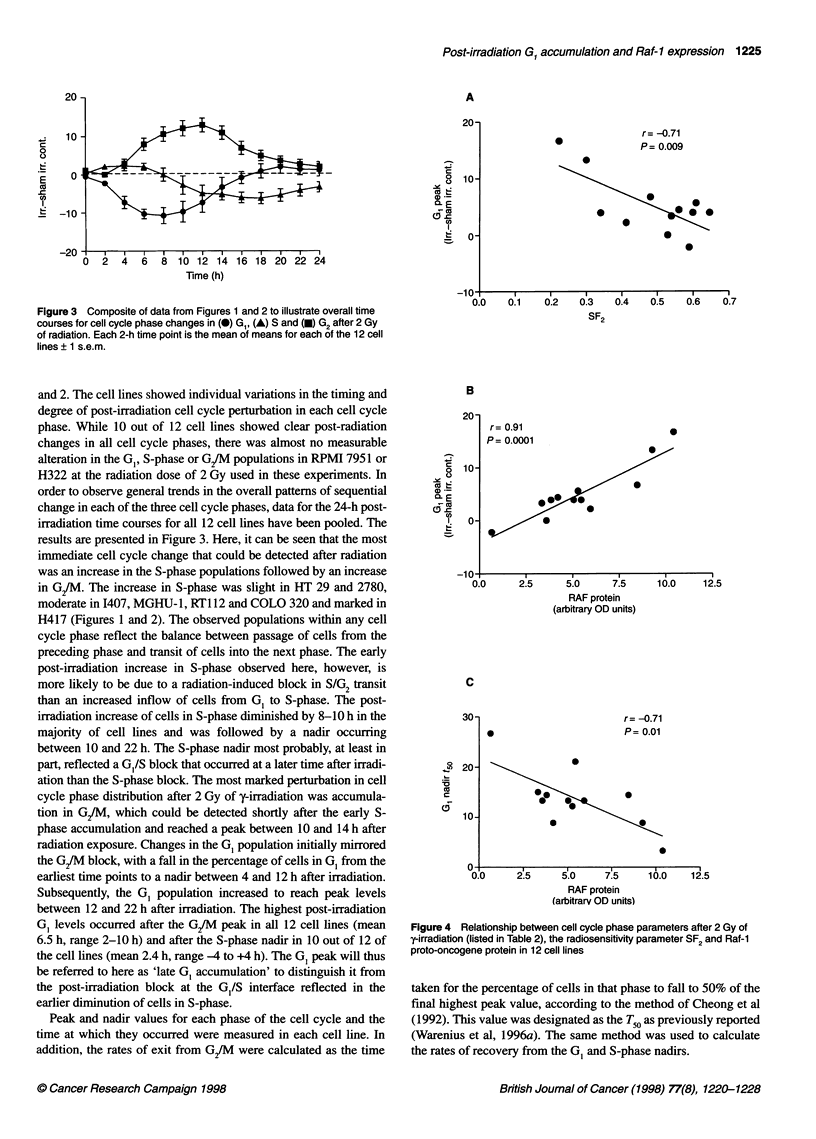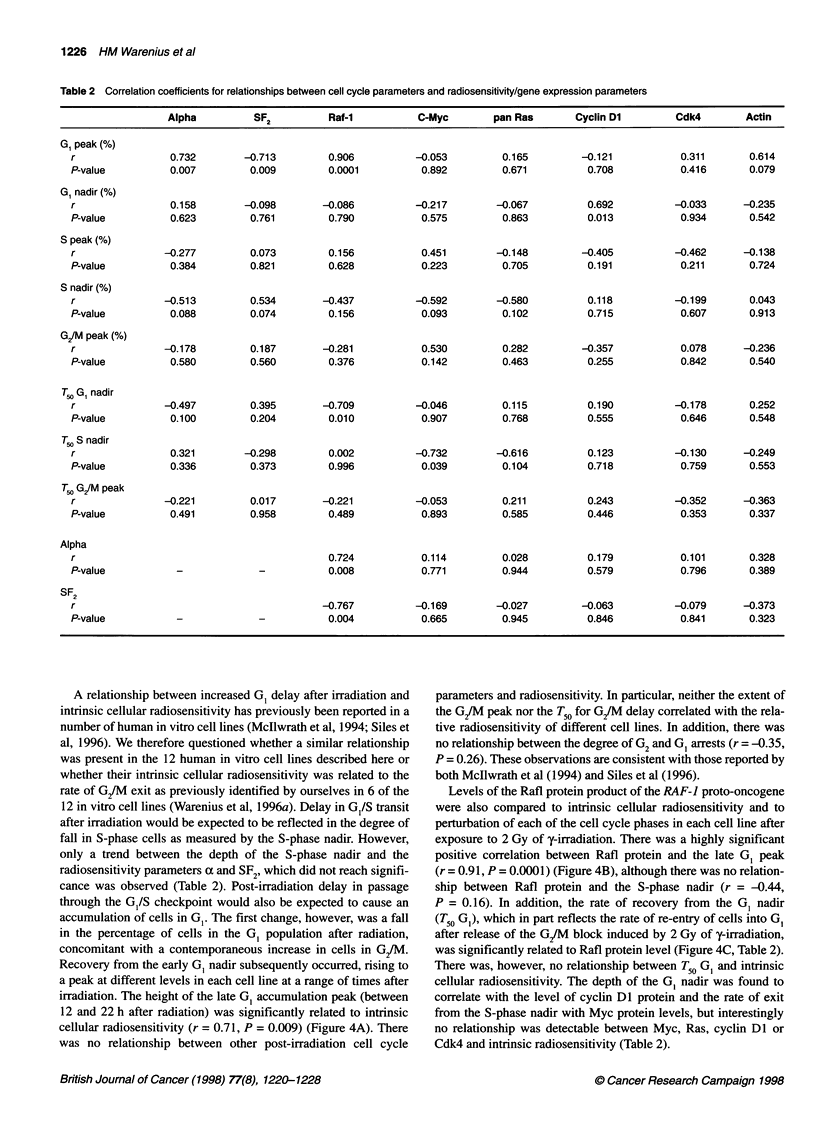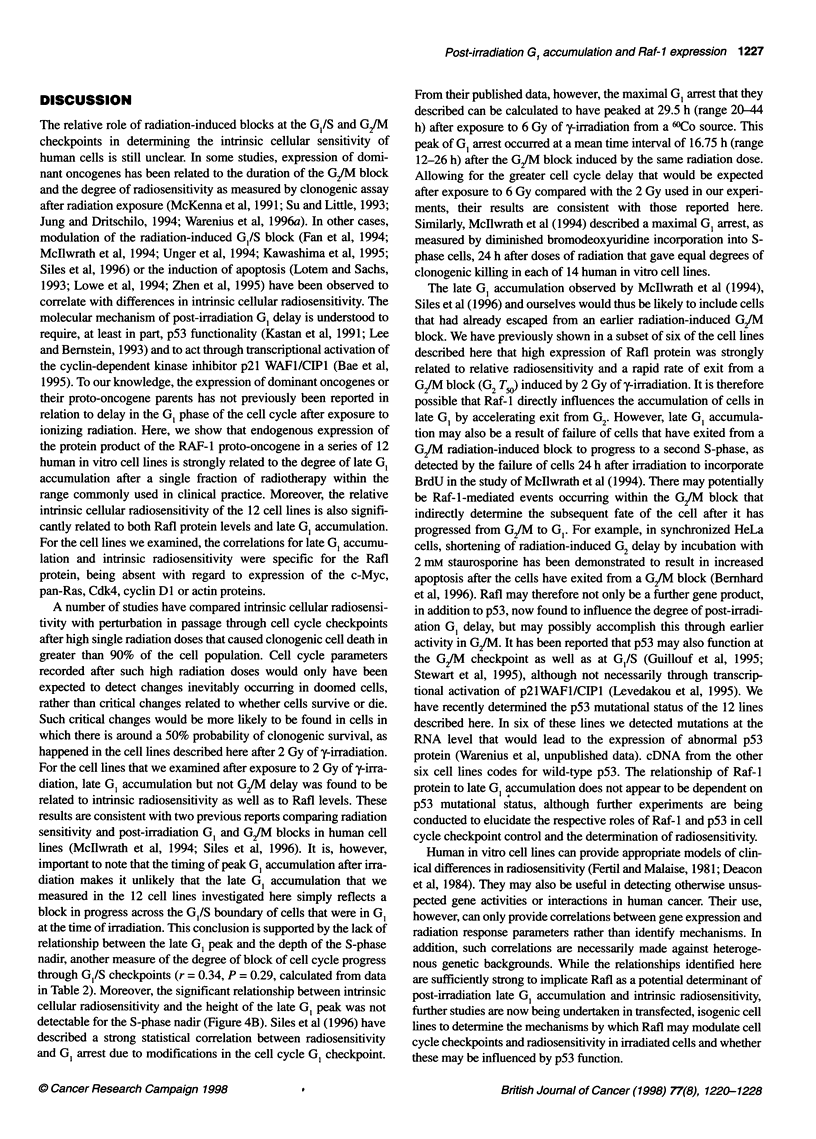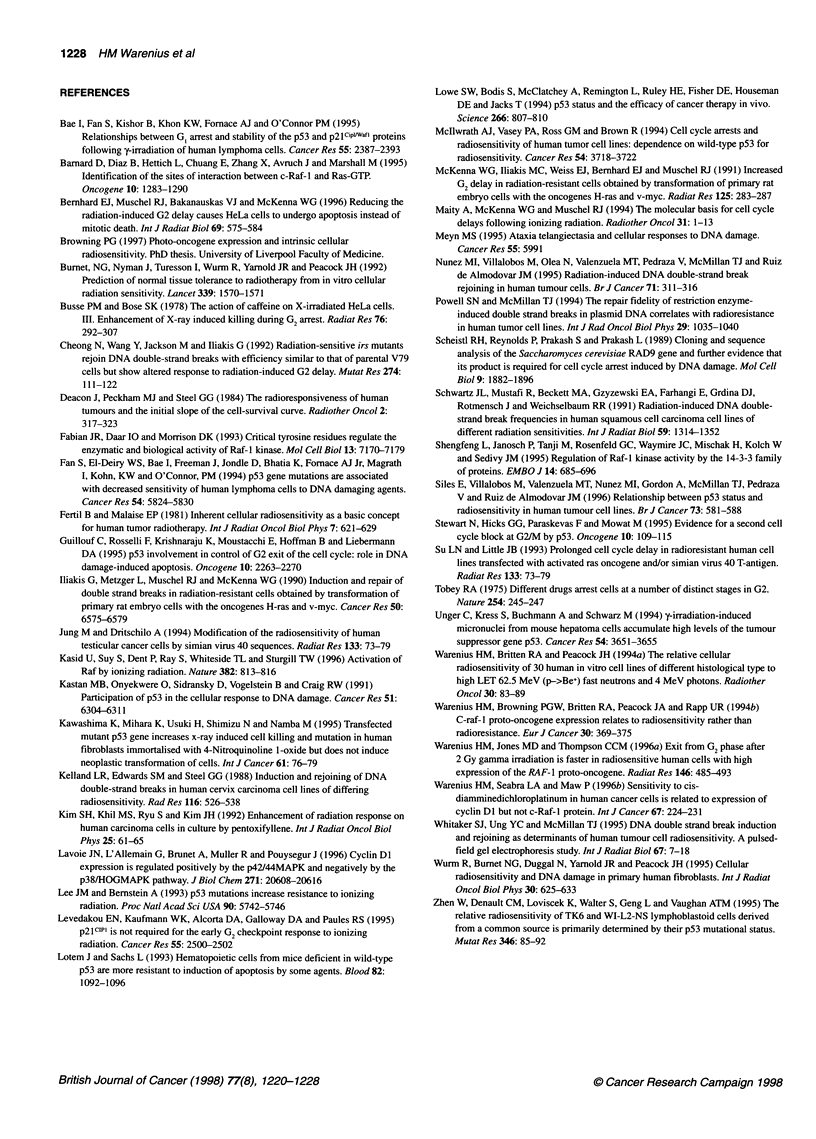# Late G1 accumulation after 2 Gy of gamma-irradiation is related to endogenous Raf-1 protein expression and intrinsic radiosensitivity in human cells.

**DOI:** 10.1038/bjc.1998.206

**Published:** 1998-04

**Authors:** H. M. Warenius, M. Jones, M. D. Jones, P. G. Browning, L. A. Seabra, C. C. Thompson

**Affiliations:** Department of Medicine, The University of Liverpool, University Clinical Departments, UK.

## Abstract

We have previously reported a correlation between high endogenous expression of the protein product of the RAF-1 proto-oncogene, intrinsic cellular radiosensitivity and rapid exit from a G2/M delay induced by 2 Gy of gamma-irradiation. Raf1 is a positive serine/threonine kinase signal transduction factor that relays signals from the cell membrane to the MAP kinase system further downstream and is believed to be involved in an ionizing radiation signal transduction pathway modulating the G1/S checkpoint. We therefore extended our flow cytometric studies to investigate relationships between radiosensitivity, endogenous expression of the Raf1 protein and perturbation of cell cycle checkpoints, leading to alterations in the G1, S and G2/M populations after 2 Gy of gamma-irradiation. Differences in intrinsic radiosensitivity after modulation of the G1/S checkpoint have generally been understood to involve p53 function up to the present time. A role for dominant oncogenes in control of G1/S transit in radiation-treated cells has not been identified previously. Here, we show in 12 human in vitro cancer cell lines that late G1 accumulation after 2 Gy of radiation is related to both Raf1 expression (r = 0.91, P = 0.0001) and the radiosensitivity parameter SF2 (r = -0.71, P = 0.009).


British Journal of Cancer (1998) 77(8), 1220–1228
© 1998 Cancer Research Campaign

### Late $G_1$ accumulation after 2 Gy of $\gamma$-irradiation is related to endogenous Raf-1 protein expression and intrinsic radiosensitivity in human cells

**HM Warenius, M Jones, MD Jones, PG Browning, LA Seabra and CCM Thompson**

Human Tumour Biology Group, Oncology Research Unit, Department of Medicine, The University of Liverpool, University Clinical Departments, The Duncan Building, Daulby Street, Liverpool L69 3GA, UK

**Summary** We have previously reported a correlation between high endogenous expression of the protein product of the *RAF-1* proto-oncogene, intrinsic cellular radiosensitivity and rapid exit from a $G_2$/M delay induced by 2 Gy of $\gamma$-irradiation. Rafl is a positive serine/threonine kinase signal transduction factor that relays signals from the cell membrane to the MAP kinase system further downstream and is believed to be involved in an ionizing radiation signal transduction pathway modulating the $G_1$/S checkpoint. We therefore extended our flow cytometric studies to investigate relationships between radiosensitivity, endogenous expression of the Rafl protein and perturbation of cell cycle checkpoints, leading to alterations in the $G_1$, S and $G_2$/M populations after 2 Gy of $\gamma$-irradiation. Differences in intrinsic radiosensitivity after modulation of the $G_1$/S checkpoint have generally been understood to involve p53 function up to the present time. A role for dominant oncogenes in control of $G_1$/S transit in radiation-treated cells has not been identified previously. Here, we show in 12 human in vitro cancer cell lines that late $G_1$ accumulation after 2 Gy of radiation is related to both Rafl expression ($r = 0.91$, $P = 0.0001$) and the radiosensitivity parameter $SF_2$ ($r = -0.71$, $P = 0.009$).

**Keywords:** irradiation; cell cycle checkpoint; $G_1$ delay; human cell line; Raf-1; oncogene

Intrinsic cellular radiosensitivity is now widely recognized as a significant factor influencing the clinical response of tumours (Fertil and Malaise, 1981; Deacon et al, 1984) and normal tissues (Burnet et al, 1992; Wurm et al, 1995) to ionizing radiation. Certain radiobiological parameters have been implicated as important determinants of the degree of intrinsic radiosensitivity shown by different cells. These include the initial damage induced by the radiation as reflected in the incidence of double-strand breaks (dsbs) in the DNA (Kelland et al, 1988; Schwartz et al, 1991), the residual damage remaining in the DNA after cellular rejoining of dsbs (Nunez et al, 1995; Whitaker et al, 1995) and the fidelity of DNA repair (Powell and McMillan, 1994). More recently, it has become increasingly apparent that, in addition to DNA damage and repair, perturbation of progress through the cell cycle consequent upon exposure to radiation may also play an important role in determining the degree of intrinsic radiosensitivity exhibited by mammalian cells.

After exposure to ionizing radiation, cell cycle progression has been observed to arrest at discrete transition points described as checkpoints (reviewed by Maity et al, 1994). These predominantly occur between $G_1$ and S-phase, during S-phase and at $G_2$/M. It has been suggested that delay in transit through cell cycle checkpoints after exposure to DNA-damaging agents allows time for repair of the DNA damage (Tobey, 1975). The degree of radiation-induced cell cycle arrest at checkpoints might thus be anticipated to influence sensitivity to cytotoxic drugs and radiation. This putative relationship has been most studied for the $G_2$/M checkpoint. In individual cell lines, shortening of the length of post-radiation $G_2$ delay as a result of mutations in genes, including the *ATM* (Meyn, 1995) and *rad9* gene (Scheistl et al, 1989), or by the drugs caffeine (Busse and Bose, 1978) and pentoxyfylline (Kim et al, 1992) can be shown to be related to increased radiosensitivity. Also transfection of normal cell lines with dominant oncogenes, such as *myc* and *ras* (McKenna et al, 1991), or SV40 (Su and Little, 1993) has resulted in increased radioresistance and a concomitant increase in $G_2$ delay even in the absence of detectable changes in the rate of dsb induction (Iliakis et al, 1990). However, a general relationship between $G_2$ delay and intrinsic radiosensitivity has not been convincingly demonstrated across the wide range of cell lines described in the literature (Warenius et al, 1996*a*).

$G_1$ delay, after exposure to ionizing radiation, has also been implicated as an important measure of cell cycle perturbation, which correlates with relative radiation sensitivity (Kastan et al, 1991; McIlwrath et al, 1994; Siles et al, 1996).

We have previously reported that high endogenous expression of Rafl, the protein product of the *RAF-1* proto-oncogene was significantly related to intrinsic cellular radiosensitivity in a series of 19 human in vitro cell lines (Warenius et al, 1994*b*). The Rafl protein was found to be strongly related to radiosensitivity and rapid exit from a radiation-induced $G_2$/M block in a subset of 7 of the 19 cell lines (Warenius et al, 1996*a*). Rafl is a serine/threonine kinase-positive signal transduction factor that relays signals from the cell membrane to the MAP kinase system further downstream. It is believed to undergo oligomerization by 14.3.3 proteins (Shengfeng et al, 1995) and binding to Ras by an interaction involving an arg89 residue in the N-terminal regulatory region of

*Received 6 May 1997*
*Revised 22 September 1997*
*Accepted 30 September 1997*

*Correspondence to:* HM Warenius the protein (Barnard et al, 1995). The 14.3.3/Rafl/Ras complex may localize at the inner cell membrane where activation is completed by phosphorylation of a Tyr 340/341 residue in Rafl by tyrosine kinases, such as Src (Fabian et al, 1993). Mek, the first member of the Mek/Erk kinase cascade binds to the C-terminal region of activated Rafl and is phosphorylated at the ATP binding site at residue lys375 in the kinase region. The Raf/Mek/Erk pathway is strongly implicated in the regulation of growth and differentiation of different cell types, and activation of this pathway has been shown to fully induce cyclin D1 (Lavoie et al, 1996), which in combination with Cdk4 or Cdk6 controls the start checkpoint in mid $G_1$. Rafl has also been shown to be tyrosine phosphorylated and activated after exposure to 15 Gy of $^{137}Cs$ γ-irradiation and to be involved in an ionizing radiation signal transduction pathway (Kasid et al, 1996). This pathway might potentially influence the response of the $G_1/S$ checkpoint to ionizing radiation. Having previously demonstrated a potential role for Rafl in control of post-irradiation exit from $G_2/M$, we now investigate its possible relationship to post-irradiation perturbation in the $G_1$ and S-phases of the cell cycle in a panel of 12 human in vitro cell lines.

### MATERIALS AND METHODS

### Cell lines and culture conditions

All of the 12 human cell lines described here (Table 1) are well established, many having been growing in vitro for several years. Tissue culture requirements, intrinsic cellular radiosensitivity (as defined by clonogenic cell survival assay) and level of the Rafl protein (as measured by Western blotting and photodensitometry) have already been described for the 12 cell lines (Warenius et al, 1994*a* and *b*). Cell lines were either donations or purchased by our laboratories. On receipt all cell lines were grown for five passages to provide sufficient cells for batch storage in liquid nitrogen. During this period, contamination was excluded by at least one passage in antibiotic-free medium, and mycoplasma testing was carried out on all lines. For clonogenic assays and preparation of lysates for quantitative Western blotting, cells were taken from a designated primary liquid nitrogen batch and grown for three to six passages until there were sufficient well-growing cells. Further batches from these cells were frozen in liquid nitrogen. The cell lines used for flow cytometry studies of perturbation in cell cycle progress after 2 Gy of γ-irradiation, described here, were freshly grown from these original batches. Two of the cell lines, OAW42 and 2780, were originally explanted from clinical ovarian carcinomas; MGHU1 and RT112 were from bladder cancers. H417 and H322 were from small-cell lung cancers, and HT29 and COLO320 from adenocarcinomas of the colon. HRT 18 was derived from an adenocarcinoma of the rectum, HEP2 from a squamous carcinoma of the larynx, RPMI from a malignant melanoma of skin and I407 was originally derived from normal embryonic intestinal epithelium but is now aneuploid.

### Effect of γ-irradiation on cell cycle progress

Perturbation in transit through the $G_1/S$ and $G_2/M$ checkpoints after exposure to 2 Gy of γ-irradiation was followed by flow cytometry (FCM). Clonogenic cell survival parameters are from previous data on cells from the same liquid nitrogen batch fitted to the linear–quadratic model as previously described (Warenius et al, 1994*a*) to yield alpha (the initial slope of the radiation cell survival curve) + 1 s.e.m. and $SF_2$ (the surviving fraction after 2 Gy of radiation) derived by interpolation of the respective fitted survival curves for each cell line. All FCM experiments were carried out on asynchronous, exponentially growing cultures as previously described (Warenius et al, 1996*a*). We chose to study perturbation of progress through $G_1/S$ and $G_2/M$ after the relatively low radiation dose of 2 Gy because we were interested in the relative effects of radiation at clinically relevant doses at which we had already demonstrated the Rafl protein level to be related to intrinsic cellular radiosensitivity (Warenius et al, 1994*b*). Also, in the 12 cell lines described here, a radiation dose of 2 Gy gave a mean clonogenic cell survival value of around 50%. Irradiation was carried out on healthy, mycoplasma-free, exponentially growing, adherent cell cultures.

### Flow cytometry

Cells were plated at a density of between $1.5 \times 10^5$ and $2 \times 10^5$ cells in 25 $cm^2$ flasks (Costar) in 8 ml of normal medium the day before irradiation. Aliquots of cells were plated in pairs of flasks for each time point. Once the cells had attached, the flasks were completely filled with medium containing 10% FCS and 20 mM HEPES and incubated at 37°C overnight. One of each pair of flasks was either sham irradiated or exposed to 2 Gy of γ-radiation from a $^{137}Cs$ Gammacell unit at 4 Gy $min^{-1}$. Care was taken to minimize any falls in temperature during this stage. After irradiation, the flasks were returned to the incubator and subsequently harvested by trypsinization at 2-h intervals, washed once in phosphate-buffered saline (PBS), fixed in 70% ethanol and stored at 4°C. For DNA content analysis, fixed cells were pelleted and washed once by centrifugation followed by resuspension in PBS containing 20 μg $ml^{-1}$ propidium iodide (PI) and 100 μg $ml^{-1}$ RNAase A. Samples were incubated for at least 30 min before being filtered through a 41-mm nylon filter (Spectrum, Texas) and then analysed on a Becton Dickinson FACS 420 flow cytometer using 488 nm illumination and a 620 nm long-pass filter. Collected list mode data were analysed using the ModfFit cell cycle analysis software (Verity, Maine). This enabled the relative distribution of cells in the $G_1$, S and $G_2/M$ phases of the cell cycle to be determined.

Three experimental runs, each recording 2-h time points for 24 h for both irradiated cells and sham-irradiated controls, were carried out for each cell line. At each 2-h time point for each run, values for $G_1$, S and $G_2/M$ obtained for sham-irradiated cells were subtracted from those for the irradiated sample. The mean of three independent runs + 1 s.e.m. was then calculated for each 2-h time point.

Data were plotted and linear regression analyses carried out using a Graphpad 2 Prism program (Graphpad, 10855 Sorrento Valley Road, San Diego, CA 92121, USA).

### Western blotting

Western blotting for the Rafl protein was carried out as previously described (Warenius et al, 1994*b*). Myc and Ras proteins were also measured because they are dominant oncogenes that have been implicated in changes in radiosensitivity and $G_2$ delay in transfected cell lines (McKenna et al, 1991). Cdk4 and cyclin D1 proteins were measured because they are pivotal molecules in the control of the start component of the $G_1/S$ checkpoint. Actin

© Cancer Research Campaign 1998

**Table 1** Radiosensitivity and gene expression parameters of 12 human cancer cell lines

| Cell lines | Histology | Radiosensitivity | | Protein expression | | | | | |
|---|---|---|---|---|---|---|---|---|---|
| | | Alpha photons | $SF_2$ | Raf-1 | Cyclin D1 | Cdk4 | C-Myc | pan Ras | Actin |
| HT29 | Adenocarcinoma colon | 0.189 (0.04) | 0.589 | 0.65 (0.18) | 9.39 (1.47) | 5.35 (0.30) | 4.32 (0.60) | 6.33 (0.60) | 1.96 (0.16) |
| I407 | Embryonic intestinal epithelium | 0.230 (0.04) | 0.539 | 3.33 (0.30) | 0.28 (0.01) | 1.96 (0.08) | 4.79 (0.21) | 3.40 (0.31) | 5.27 (0.07) |
| MGHU-1 | Transition cell carcinoma bladder | 0.200 (0.06) | 0.608 | 5.27 (0.67) | 6.23 (0.28) | 4.07 (0.19) | 4.08 (0.73) | 7.49 (0.64) | 4.48 (0.56) |
| OAW42 | Ovarian carcinoma | 0.705 (0.07) | 0.222 | 10.39 (0.16) | 6.65 | 6.38 (0.12) | 3.63 (0.93) | 4.56 (0.23) | 5.29 (1.15) |
| RPMI 7951 | Melanoma | 0.111 (0.03) | 0.56 | 4.19 (0.43) | 4.79 | – | – | – | – |
| RT112 | Transition cell carcinoma bladder | 0.210 (0.10) | 0.599 | 5.04 (0.91) | 3.19 (0.06) | 9.00 (0.39) | 7.11 (0.44) | 5.20 (0.19) | 4.97 (0.18) |
| 2780 | Ovarian carcinoma | 0.525 (0.05) | 0.299 | 9.25 (1.16) | 4.42 (0.06) | 7.25 (0.46) | 7.25 (0.79) | 6.56 (0.34) | 7.89 (0.51) |
| COLO320 | Adenocarcinoma colon | 0.334 (0.06) | 0.34 | 5.44 (0.90) | 4.40 | – | – | – | – |
| H322 | Small-cell carcinoma lung | 0.280 (0.03) | 0.529 | 3.58 (0.17) | 7.29 (0.62) | 3.40 (0.18) | 4.00 (0.25) | 2.17 (0.33) | 5.50 (0.06) |
| H417 | Small-cell lung carcinoma | 0.189 (0.03) | 0.48 | 8.45 (1.03) | 3.21 | – | – | – | – |
| HEP2 | Squamous carcinoma larynx | 0.113 (0.03) | 0.646 | 3.82 (0.65) | 3.35 (0.50) | 8.16 (0.19) | 5.29 (0.75) | 4.72 (0.77) | 6.05 (0.25) |
| HRT18 | Adenocarcinoma rectum | 0.411 (0.04) | 0.412 | 5.94 (0.38) | 4.44 (0.01) | 5.46 (0.62) | 10.05 (0.76) | 6.07 (0.35) | 3.90 (0.11) |

provided a non-cell proliferation-related, control protein. Then, $3 \times 10^7$ cells were plated in 162 $cm^2$ tissue culture flasks (Costar, High Wycombe, Bucks) and allowed to grow under exponential conditions for 5 days, at which time they were preconfluent and still growing exponentially as confirmed by visual observation and flow cytometry. Cells were then removed by trypsinization, resuspended in complete medium + 10% FCS to inactivate trypsin and washed three times by serial centrifugation and resuspension in PBS without serum. Approximately 1–3 $\times 10^8$ viable cells were then pelleted by centrifugation and the pellet resuspended at a concentration of $3\times10^7$ cells per 1.0 ml of lysate buffer [stock solution: 10% sodium dodecyl sulphate (SDS) 10 ml, 0.5 M Tris, pH 6.8, glycerol 10 ml, double distilled water 62 ml; to 10 ml of stock solution were added 100 ml of 10 mM Leupeptin + 10 ml of 100 mM phenylmethylsulphonyl fluoride (PMSF)]. Protein estimations were performed and the final concentration of the lysates adjusted to 150 mg of total cellular protein per 100 ml.

Rafl, Myc, Ras, Cdk4, Cyclin D1 and Actin proteins were analysed by SDS-PAGE electrophoresis as previously described (Warenius et al, 1994*b*, 1996*b*). Two independent Western immunoblottings using separately prepared lysates for each cell line loaded in pairs on each electrophoretic gel were carried out. Conditions were optimized for each protein measured, and linearity studies at different protein loadings were made to confirm quantitation on Western blotting (Warenius et al, 1996*b*; Browning, 1997). Then, 50–150 mg of total cellular protein in 50 ml of lysate buffer were added per lane well to 7.5–15% Laemmli separating gels as appropriate for each of the relevant proteins, and electrophoresis was carried out at 16°C using 60 V over 16 h and a constant current of 500 mA. Electrophoretic transfer of peptide bands to nitrocellulose was performed at a constant current of 500 mA for 3–6 h. The nitrocellulose blots were washed in Tris-buffered saline with 0.5% fetal calf serum (FCS) and then exposed to the following antisera: Rafl, URP 26S3 (a gift from Dr U Rapp, Wurtzburg, Germany) at 1:750; Myc, 9E10 at 1:1000 (a gift from Dr G Evan, ICRF, London); pan Ras, OP22 at 1:200 (Oncogene Science, NY, USA); Actin at 1:750 000 (ICN Biomedicals, High Wycombe, UK); Cdk4, a rabbit polyclonal antiserum at 1:250 (Santa Cruz Biotechnology, UK) and Cyclin D1, a mouse IgG1 MAb to mammalian cyclins D1, D2, D3 at 2 mg $ml^{-1}$ (G124–259.5, Pharmingen, San Diego, CA, USA). The immunoblots were thoroughly rinsed and then incubated with rabbit anti-mouse or goat anti-rabbit alkaline phosphatase-conjugated antibodies as appropriate (Dako, UK) at 1:1000 in Tris-buffered saline plus 5% FCS for 1 h at room temperature in darkness and developed in alkaline phophatase buffer containing nitroblue tetrazolium and 5-bromo-4-chloro-3-indoyl phosphate (Sigma, Poole, Dorset, UK) (50 mg $ml^{-1}$ in dimethylformamide). Quantitation of proteins was carried out by measurement of optical density on a Schimadzu scanning densitometer with tungsten light and expressed as OD units per 150 mg of total cellular protein. In order to compare different protein levels between the cell lines, the mean OD value was calculated, and the relative OD for the relevant protein in each individual cell line was normalized to its mean OD and multiplied by an arbitrary value of 5.0.

### RESULTS

The time courses of change in the $G_1$, S- and $G_2$/M phases of the cell cycle measured serially at 2-h intervals after 2 Gy of γ-irradiation for each of the 12 human in vitro cell lines are shown in Figures 1

© Cancer Research Campaign 1998

**Figure 1** Percentages of cells in the $G_1$, S- and $G_2$/M phases of the cell cycle over 24 h obtained by analysis of serial PI histograms, after exposure to 2 Gy of γ-irradiation (at time 0). For each 2-h time point, the value of the sham-irradiated cells was subtracted from that of its irradiated counterpart and the mean ± 1 s.e.m. of a minimum of three consecutive experiments was calculated. ●, Cells in $G_1$ phase; ◇, cells in S-phase; □, cells in $G_2$ phase

© Cancer Research Campaign 1998

**Figure 2** Percentages of cells in the $G_1$, S- and $G_2$/M phases of the cell cycle over 24 h obtained by analysis of serial PI histograms, after exposure to 2 Gy of γ-irradiation (at time 0). For each 2-h time point, the value of the sham-irradiated cells was subtracted from that of its irradiated counterpart and the mean ± s.e.m. of a minimum of three consecutive experiments was calculated. ●, Cells in $G_1$ phase; ◇, cells in S-phase; □, cells in $G_2$ phase

© Cancer Research Campaign 1998

**Figure 3** Composite of data from Figures 1 and 2 to illustrate overall time courses for cell cycle phase changes in (●) $G_1$, (▲) S and (■) $G_2$ after 2 Gy of radiation. Each 2-h time point is the mean of means for each of the 12 cell lines ± 1 s.e.m.

and 2. The cell lines showed individual variations in the timing and degree of post-irradiation cell cycle perturbation in each cell cycle phase. While 10 out of 12 cell lines showed clear post-radiation changes in all cell cycle phases, there was almost no measurable alteration in the $G_1$, S-phase or $G_2$/M populations in RPMI 7951 or H322 at the radiation dose of 2 Gy used in these experiments. In order to observe general trends in the overall patterns of sequential change in each of the three cell cycle phases, data for the 24-h post-irradiation time courses for all 12 cell lines have been pooled. The results are presented in Figure 3. Here, it can be seen that the most immediate cell cycle change that could be detected after radiation was an increase in the S-phase populations followed by an increase in $G_2$/M. The increase in S-phase was slight in HT 29 and 2780, moderate in I407, MGHU-1, RT112 and COLO 320 and marked in H417 (Figures 1 and 2). The observed populations within any cell cycle phase reflect the balance between passage of cells from the preceding phase and transit of cells into the next phase. The early post-irradiation increase in S-phase observed here, however, is more likely to be due to a radiation-induced block in S/$G_2$ transit than an increased inflow of cells from $G_1$ to S-phase. The post-irradiation increase of cells in S-phase diminished by 8–10 h in the majority of cell lines and was followed by a nadir occurring between 10 and 22 h. The S-phase nadir most probably, at least in part, reflected a $G_1$/S block that occurred at a later time after irradiation than the S-phase block. The most marked perturbation in cell cycle phase distribution after 2 Gy of γ-irradiation was accumulation in $G_2$/M, which could be detected shortly after the early S-phase accumulation and reached a peak between 10 and 14 h after radiation exposure. Changes in the $G_1$ population initially mirrored the $G_2$/M block, with a fall in the percentage of cells in $G_1$ from the earliest time points to a nadir between 4 and 12 h after irradiation. Subsequently, the $G_1$ population increased to reach peak levels between 12 and 22 h after irradiation. The highest post-irradiation $G_1$ levels occurred after the $G_2$/M peak in all 12 cell lines (mean 6.5 h, range 2–10 h) and after the S-phase nadir in 10 out of 12 of the cell lines (mean 2.4 h, range –4 to +4 h). The $G_1$ peak will thus be referred to here as 'late $G_1$ accumulation' to distinguish it from the post-irradiation block at the $G_1$/S interface reflected in the earlier diminution of cells in S-phase.

Peak and nadir values for each phase of the cell cycle and the time at which they occurred were measured in each cell line. In addition, the rates of exit from $G_2$/M were calculated as the time taken for the percentage of cells in that phase to fall to 50% of the final highest peak value, according to the method of Cheong et al (1992). This value was designated as the $T_{50}$ as previously reported (Warenius et al, 1996*a*). The same method was used to calculate the rates of recovery from the $G_1$ and S-phase nadirs.

**Figure 4** Relationship between cell cycle phase parameters after 2 Gy of γ-irradiation (listed in Table 2), the radiosensitivity parameter $SF_2$ and Raf-1 proto-oncogene protein in 12 cell lines

**Table 2** Correlation coefficients for relationships between cell cycle parameters and radiosensitivity/gene expression parameters

| | Alpha | $SF_2$ | Raf-1 | C-Myc | pan Ras | Cyclin D1 | Cdk4 | Actin |
|---|---|---|---|---|---|---|---|---|
| $G_1$ peak (%) | | | | | | | | |
| *r* | 0.732 | –0.713 | 0.906 | –0.053 | 0.165 | –0.121 | 0.311 | 0.614 |
| *P*-value | 0.007 | 0.009 | 0.0001 | 0.892 | 0.671 | 0.708 | 0.416 | 0.079 |
| $G_1$ nadir (%) | | | | | | | | |
| *r* | 0.158 | –0.098 | –0.086 | –0.217 | –0.067 | 0.692 | –0.033 | –0.235 |
| *P*-value | 0.623 | 0.761 | 0.790 | 0.575 | 0.863 | 0.013 | 0.934 | 0.542 |
| S peak (%) | | | | | | | | |
| *r* | –0.277 | 0.073 | 0.156 | 0.451 | –0.148 | –0.405 | –0.462 | –0.138 |
| *P*-value | 0.384 | 0.821 | 0.628 | 0.223 | 0.705 | 0.191 | 0.211 | 0.724 |
| S nadir (%) | | | | | | | | |
| *r* | –0.513 | 0.534 | –0.437 | –0.592 | –0.580 | 0.118 | –0.199 | 0.043 |
| *P*-value | 0.088 | 0.074 | 0.156 | 0.093 | 0.102 | 0.715 | 0.607 | 0.913 |
| $G_2$/M peak (%) | | | | | | | | |
| *r* | –0.178 | 0.187 | –0.281 | 0.530 | 0.282 | –0.357 | 0.078 | –0.236 |
| *P*-value | 0.580 | 0.560 | 0.376 | 0.142 | 0.463 | 0.255 | 0.842 | 0.540 |
| $T_{50}$ $G_1$ nadir | | | | | | | | |
| *r* | –0.497 | 0.395 | –0.709 | –0.046 | 0.115 | 0.190 | –0.178 | 0.252 |
| *P*-value | 0.100 | 0.204 | 0.010 | 0.907 | 0.768 | 0.555 | 0.646 | 0.548 |
| $T_{50}$ S nadir | | | | | | | | |
| *r* | 0.321 | –0.298 | 0.002 | –0.732 | –0.616 | 0.123 | –0.130 | –0.249 |
| *P*-value | 0.336 | 0.373 | 0.996 | 0.039 | 0.104 | 0.718 | 0.759 | 0.553 |
| $T_{50}$ $G_2$/M peak | | | | | | | | |
| *r* | –0.221 | 0.017 | –0.221 | –0.053 | 0.211 | 0.243 | –0.352 | –0.363 |
| *P*-value | 0.491 | 0.958 | 0.489 | 0.893 | 0.585 | 0.446 | 0.353 | 0.337 |
| Alpha | | | | | | | | |
| *r* | | | 0.724 | 0.114 | 0.028 | 0.179 | 0.101 | 0.328 |
| *P*-value | – | – | 0.008 | 0.771 | 0.944 | 0.579 | 0.796 | 0.389 |
| $SF_2$ | | | | | | | | |
| *r* | | | –0.767 | –0.169 | –0.027 | –0.063 | –0.079 | –0.373 |
| *P*-value | – | – | 0.004 | 0.665 | 0.945 | 0.846 | 0.841 | 0.323 |

A relationship between increased $G_1$ delay after irradiation and intrinsic cellular radiosensitivity has previously been reported in a number of human in vitro cell lines (McIlwrath et al, 1994; Siles et al, 1996). We therefore questioned whether a similar relationship was present in the 12 human in vitro cell lines described here or whether their intrinsic cellular radiosensitivity was related to the rate of $G_2$/M exit as previously identified by ourselves in 6 of the 12 in vitro cell lines (Warenius et al, 1996*a*). Delay in $G_1$/S transit after irradiation would be expected to be reflected in the degree of fall in S-phase cells as measured by the S-phase nadir. However, only a trend between the depth of the S-phase nadir and the radiosensitivity parameters α and $SF_2$, which did not reach significance was observed (Table 2). Post-irradiation delay in passage through the $G_1$/S checkpoint would also be expected to cause an accumulation of cells in $G_1$. The first change, however, was a fall in the percentage of cells in the $G_1$ population after radiation, concomitant with a contemporaneous increase in cells in $G_2$/M. Recovery from the early $G_1$ nadir subsequently occurred, rising to a peak at different levels in each cell line at a range of times after irradiation. The height of the late $G_1$ accumulation peak (between 12 and 22 h after radiation) was significantly related to intrinsic cellular radiosensitivity ($r$ = 0.71, $P$ = 0.009) (Figure 4A). There was no relationship between other post-irradiation cell cycle parameters and radiosensitivity. In particular, neither the extent of the $G_2$/M peak nor the $T_{50}$ for $G_2$/M delay correlated with the relative radiosensitivity of different cell lines. In addition, there was no relationship between the degree of $G_2$ and $G_1$ arrests ($r$ = –0.35, $P$ = 0.26). These observations are consistent with those reported by both McIlwrath et al (1994) and Siles et al (1996).

Levels of the Rafl protein product of the *RAF-1* proto-oncogene were also compared to intrinsic cellular radiosensitivity and to perturbation of each of the cell cycle phases in each cell line after exposure to 2 Gy of γ-irradiation. There was a highly significant positive correlation between Rafl protein and the late $G_1$ peak ($r$ = 0.91, $P$ = 0.0001) (Figure 4B), although there was no relationship between Rafl protein and the S-phase nadir ($r$ = –0.44, $P$ = 0.16). In addition, the rate of recovery from the $G_1$ nadir ($T_{50}$ $G_1$), which in part reflects the rate of re-entry of cells into $G_1$ after release of the $G_2$/M block induced by 2 Gy of γ-irradiation, was significantly related to Rafl protein level (Figure 4C, Table 2). There was, however, no relationship between $T_{50}$ $G_1$ and intrinsic cellular radiosensitivity. The depth of the $G_1$ nadir was found to correlate with the level of cyclin D1 protein and the rate of exit from the S-phase nadir with Myc protein levels, but interestingly no relationship was detectable between Myc, Ras, cyclin D1 or Cdk4 and intrinsic radiosensitivity (Table 2).

© Cancer Research Campaign 1998

### DISCUSSION

The relative role of radiation-induced blocks at the $G_1$/S and $G_2$/M checkpoints in determining the intrinsic cellular sensitivity of human cells is still unclear. In some studies, expression of dominant oncogenes has been related to the duration of the $G_2$/M block and the degree of radiosensitivity as measured by clonogenic assay after radiation exposure (McKenna et al, 1991; Su and Little, 1993; Jung and Dritschilo, 1994; Warenius et al, 1996*a*). In other cases, modulation of the radiation-induced $G_1$/S block (Fan et al, 1994; McIlwrath et al, 1994; Unger et al, 1994; Kawashima et al, 1995; Siles et al, 1996) or the induction of apoptosis (Lotem and Sachs, 1993; Lowe et al, 1994; Zhen et al, 1995) have been observed to correlate with differences in intrinsic cellular radiosensitivity. The molecular mechanism of post-irradiation $G_1$ delay is understood to require, at least in part, p53 functionality (Kastan et al, 1991; Lee and Bernstein, 1993) and to act through transcriptional activation of the cyclin-dependent kinase inhibitor p21 WAF1/CIP1 (Bae et al, 1995). To our knowledge, the expression of dominant oncogenes or their proto-oncogene parents has not previously been reported in relation to delay in the $G_1$ phase of the cell cycle after exposure to ionizing radiation. Here, we show that endogenous expression of the protein product of the RAF-1 proto-oncogene in a series of 12 human in vitro cell lines is strongly related to the degree of late $G_1$ accumulation after a single fraction of radiotherapy within the range commonly used in clinical practice. Moreover, the relative intrinsic cellular radiosensitivity of the 12 cell lines is also significantly related to both Rafl protein levels and late $G_1$ accumulation. For the cell lines we examined, the correlations for late $G_1$ accumulation and intrinsic radiosensitivity were specific for the Rafl protein, being absent with regard to expression of the c-Myc, pan-Ras, Cdk4, cyclin D1 or actin proteins.

A number of studies have compared intrinsic cellular radiosensitivity with perturbation in passage through cell cycle checkpoints after high single radiation doses that caused clonogenic cell death in greater than 90% of the cell population. Cell cycle parameters recorded after such high radiation doses would only have been expected to detect changes inevitably occurring in doomed cells, rather than critical changes related to whether cells survive or die. Such critical changes would be more likely to be found in cells in which there is around a 50% probability of clonogenic survival, as happened in the cell lines described here after 2 Gy of $\gamma$-irradiation. For the cell lines that we examined after exposure to 2 Gy of $\gamma$-irradiation, late $G_1$ accumulation but not $G_2$/M delay was found to be related to intrinsic radiosensitivity as well as to Rafl levels. These results are consistent with two previous reports comparing radiation sensitivity and post-irradiation $G_1$ and $G_2$/M blocks in human cell lines (McIlwrath et al, 1994; Siles et al, 1996). It is, however, important to note that the timing of peak $G_1$ accumulation after irradiation makes it unlikely that the late $G_1$ accumulation that we measured in the 12 cell lines investigated here simply reflects a block in progress across the $G_1$/S boundary of cells that were in $G_1$ at the time of irradiation. This conclusion is supported by the lack of relationship between the late $G_1$ peak and the depth of the S-phase nadir, another measure of the degree of block of cell cycle progress through $G_1$/S checkpoints ($r = 0.34$, $P = 0.29$, calculated from data in Table 2). Moreover, the significant relationship between intrinsic cellular radiosensitivity and the height of the late $G_1$ peak was not detectable for the S-phase nadir (Figure 4B). Siles et al (1996) have described a strong statistical correlation between radiosensitivity and $G_1$ arrest due to modifications in the cell cycle $G_1$ checkpoint. From their published data, however, the maximal $G_1$ arrest that they described can be calculated to have peaked at 29.5 h (range 20–44 h) after exposure to 6 Gy of $\gamma$-irradiation from a $^{60}$Co source. This peak of $G_1$ arrest occurred at a mean time interval of 16.75 h (range 12–26 h) after the $G_2$/M block induced by the same radiation dose. Allowing for the greater cell cycle delay that would be expected after exposure to 6 Gy compared with the 2 Gy used in our experiments, their results are consistent with those reported here. Similarly, McIlwrath et al (1994) described a maximal $G_1$ arrest, as measured by diminished bromodeoxyuridine incorporation into S-phase cells, 24 h after doses of radiation that gave equal degrees of clonogenic killing in each of 14 human in vitro cell lines.

The late $G_1$ accumulation observed by McIlwrath et al (1994), Siles et al (1996) and ourselves would thus be likely to include cells that had already escaped from an earlier radiation-induced $G_2$/M block. We have previously shown in a subset of six of the cell lines described here that high expression of Rafl protein was strongly related to relative radiosensitivity and a rapid rate of exit from a $G_2$/M block ($G_2$ $T_{50}$) induced by 2 Gy of $\gamma$-irradiation. It is therefore possible that Raf-1 directly influences the accumulation of cells in late $G_1$ by accelerating exit from $G_2$. However, late $G_1$ accumulation may also be a result of failure of cells that have exited from a $G_2$/M radiation-induced block to progress to a second S-phase, as detected by the failure of cells 24 h after irradiation to incorporate BrdU in the study of McIlwrath et al (1994). There may potentially be Raf-1-mediated events occurring within the $G_2$/M block that indirectly determine the subsequent fate of the cell after it has progressed from $G_2$/M to $G_1$. For example, in synchronized HeLa cells, shortening of radiation-induced $G_2$ delay by incubation with 2 mM staurosporine has been demonstrated to result in increased apoptosis after the cells have exited from a $G_2$/M block (Bernhard et al, 1996). Rafl may therefore not only be a further gene product, in addition to p53, now found to influence the degree of post-irradiation $G_1$ delay, but may possibly accomplish this through earlier activity in $G_2$/M. It has been reported that p53 may also function at the $G_2$/M checkpoint as well as at $G_1$/S (Guillouf et al, 1995; Stewart et al, 1995), although not necessarily through transcriptional activation of p21WAF1/CIP1 (Levedakou et al, 1995). We have recently determined the p53 mutational status of the 12 lines described here. In six of these lines we detected mutations at the RNA level that would lead to the expression of abnormal p53 protein (Warenius et al, unpublished data). cDNA from the other six cell lines codes for wild-type p53. The relationship of Raf-1 protein to late $G_1$ accumulation does not appear to be dependent on p53 mutational status, although further experiments are being conducted to elucidate the respective roles of Raf-1 and p53 in cell cycle checkpoint control and the determination of radiosensitivity.

Human in vitro cell lines can provide appropriate models of clinical differences in radiosensitivity (Fertil and Malaise, 1981; Deacon et al, 1984). They may also be useful in detecting otherwise unsuspected gene activities or interactions in human cancer. Their use, however, can only provide correlations between gene expression and radiation response parameters rather than identify mechanisms. In addition, such correlations are necessarily made against heterogenous genetic backgrounds. While the relationships identified here are sufficiently strong to implicate Rafl as a potential determinant of post-irradiation late $G_1$ accumulation and intrinsic radiosensitivity, further studies are now being undertaken in transfected, isogenic cell lines to determine the mechanisms by which Rafl may modulate cell cycle checkpoints and radiosensitivity in irradiated cells and whether these may be influenced by p53 function.

© Cancer Research Campaign 1998

### REFERENCES

Bae I, Fan S, Kishor B, Khon KW, Fornace AJ and O'Connor PM (1995) Relationships between $G_1$ arrest and stability of the p53 and p21$^{Cipl/Waf1}$ proteins following γ-irradiation of human lymphoma cells. *Cancer Res* **55**: 2387–2393

Barnard D, Diaz B, Hettich L, Chuang E, Zhang X, Avruch J and Marshall M (1995) Identification of the sites of interaction between c-Raf-1 and Ras-GTP. *Oncogene* **10**: 1283–1290

Bernhard EJ, Muschel RJ, Bakanauskas VJ and McKenna WG (1996) Reducing the radiation-induced G2 delay causes HeLa cells to undergo apoptosis instead of mitotic death. *Int J Radiat Biol* **69**: 575–584

Browning PG (1997) Photo-oncogene expression and intrinsic cellular radiosensitivity. PhD thesis. University of Liverpool Faculty of Medicine.

Burnet, NG, Nyman J, Turesson I, Wurm R, Yarnold JR and Peacock JH (1992) Prediction of normal tissue tolerance to radiotherapy from in vitro cellular radiation sensitivity. *Lancet* **339**: 1570–1571

Busse PM and Bose SK (1978) The action of caffeine on X-irradiated HeLa cells. III. Enhancement of X-ray induced killing during $G_2$ arrest. *Radiat Res* **76**: 292–307

Cheong N, Wang Y, Jackson M and Iliakis G (1992) Radiation-sensitive *irs* mutants rejoin DNA double-strand breaks with efficiency similar to that of parental V79 cells but show altered response to radiation-induced G2 delay. *Mutat Res* **274**: 111–122

Deacon J, Peckham MJ and Steel GG (1984) The radioresponsiveness of human tumours and the initial slope of the cell-survival curve. *Radiother Oncol* **2**: 317–323

Fabian JR, Daar IO and Morrison DK (1993) Critical tyrosine residues regulate the enzymatic and biological activity of Raf-1 kinase. *Mol Cell Biol* **13**: 7170–7179

Fan S, El-Deiry WS, Bae I, Freeman J, Jondle D, Bhatia K, Fornace AJ Jr, Magrath I, Kohn, KW and O'Connor, PM (1994) p53 gene mutations are associated with decreased sensitivity of human lymphoma cells to DNA damaging agents. *Cancer Res* **54**: 5824–5830

Fertil B and Malaise EP (1981) Inherent cellular radiosensitivity as a basic concept for human tumor radiotherapy. *Int J Radiat Oncol Biol Phys* **7**: 621–629

Guillouf C, Rosselli F, Krishnaraju K, Moustacchi E, Hoffman B and Liebermann DA (1995) p53 involvement in control of G2 exit of the cell cycle: role in DNA damage-induced apoptosis. *Oncogene* **10**: 2263–2270

Iliakis G, Metzger L, Muschel RJ and McKenna WG (1990) Induction and repair of double strand breaks in radiation-resistant cells obtained by transformation of primary rat embryo cells with the oncogenes H-ras and v-myc. *Cancer Res* **50**: 6575–6579

Jung M and Dritschilo A (1994) Modification of the radiosensitivity of human testicular cancer cells by simian virus 40 sequences. *Radiat Res* **133**: 73–79

Kasid U, Suy S, Dent P, Ray S, Whiteside TL and Sturgill TW (1996) Activation of Raf by ionizing radiation. *Nature* **382**: 813–816

Kastan MB, Onyekwere O, Sidransky D, Vogelstein B and Craig RW (1991) Participation of p53 in the cellular response to DNA damage. *Cancer Res* **51**: 6304–6311

Kawashima K, Mihara K, Usuki H, Shimizu N and Namba M (1995) Transfected mutant p53 gene increases x-ray induced cell killing and mutation in human fibroblasts immortalised with 4-Nitroquinoline 1-oxide but does not induce neoplastic transformation of cells. *Int J Cancer* **61**: 76–79

Kelland LR, Edwards SM and Steel GG (1988) Induction and rejoining of DNA double-strand breaks in human cervix carcinoma cell lines of differing radiosensitivity. *Rad Res* **116**: 526–538

Kim SH, Khil MS, Ryu S and Kim JH (1992) Enhancement of radiation response on human carcinoma cells in culture by pentoxifyllene. *Int J Radiat Oncol Biol Phys* **25**: 61–65

Lavoie JN, L'Allemain G, Brunet A, Muller R and Pouysegur J (1996) Cyclin D1 expression is regulated positively by the p42/44MAPK and negatively by the p38/HOGMAPK pathway. *J Biol Chem* **271**: 20608–20616

Lee JM and Bernstein A (1993) p53 mutations increase resistance to ionizing radiation. *Proc Natl Acad Sci USA* **90**: 5742–5746

Levedakou EN, Kaufmann WK, Alcorta DA, Galloway DA and Paules RS (1995) p21$^{CIP1}$ is not required for the early $G_2$ checkpoint response to ionizing radiation. *Cancer Res* **55**: 2500–2502

Lotem J and Sachs L (1993) Hematopoietic cells from mice deficient in wild-type p53 are more resistant to induction of apoptosis by some agents. *Blood* **82**: 1092–1096

Lowe SW, Bodis S, McClatchey A, Remington L, Ruley HE, Fisher DE, Houseman DE and Jacks T (1994) p53 status and the efficacy of cancer therapy in vivo. *Science* **266**: 807–810

McIlwrath AJ, Vasey PA, Ross GM and Brown R (1994) Cell cycle arrests and radiosensitivity of human tumor cell lines: dependence on wild-type p53 for radiosensitivity. *Cancer Res* **54**: 3718–3722

McKenna WG, Iliakis MC, Weiss EJ, Bernhard EJ and Muschel RJ (1991) Increased $G_2$ delay in radiation-resistant cells obtained by transformation of primary rat embryo cells with the oncogenes H-ras and v-myc. *Radiat Res* **125**: 283–287

Maity A, McKenna WG and Muschel RJ (1994) The molecular basis for cell cycle delays following ionizing radiation. *Radiother Oncol* **31**: 1–13

Meyn MS (1995) Ataxia telangiectasia and cellular responses to DNA damage. *Cancer Res* **55**: 5991

Nunez MI, Villalobos M, Olea N, Valenzuela MT, Pedraza V, McMillan TJ and Ruiz de Almodovar JM (1995) Radiation-induced DNA double-strand break rejoining in human tumour cells. *Br J Cancer* **71**: 311–316

Powell SN and McMillan TJ (1994) The repair fidelity of restriction enzyme-induced double strand breaks in plasmid DNA correlates with radioresistance in human tumor cell lines. *Int J Rad Oncol Biol Phys* **29**: 1035–1040

Scheistl RH, Reynolds P, Prakash S and Prakash L (1989) Cloning and sequence analysis of the *Saccharomyces cerevisiae* RAD9 gene and further evidence that its product is required for cell cycle arrest induced by DNA damage. *Mol Cell Biol* **9**: 1882–1896

Schwartz JL, Mustafi R, Beckett MA, Gzyzewski EA, Farhangi E, Grdina DJ, Rotmensch J and Weichselbaum RR (1991) Radiation-induced DNA double-strand break frequencies in human squamous cell carcinoma cell lines of different radiation sensitivities. *Int J Radiat Biol* **59**: 1314–1352

Shengfeng L, Janosch P, Tanji M, Rosenfeld GC, Waymire JC, Mischak H, Kolch W and Sedivy JM (1995) Regulation of Raf-1 kinase activity by the 14-3-3 family of proteins. *EMBO J* **14**: 685–696

Siles E, Villalobos M, Valenzuela MT, Nunez MI, Gordon A, McMillan TJ, Pedraza V and Ruiz de Almodovar JM (1996) Relationship between p53 status and radiosensitivity in human tumour cell lines. *Br J Cancer* **73**: 581–588

Stewart N, Hicks GG, Paraskevas F and Mowat M (1995) Evidence for a second cell cycle block at G2/M by p53. *Oncogene* **10**: 109–115

Su LN and Little JB (1993) Prolonged cell cycle delay in radioresistant human cell lines transfected with activated ras oncogene and/or simian virus 40 T-antigen. *Radiat Res* **133**: 73–79

Tobey RA (1975) Different drugs arrest cells at a number of distinct stages in G2. *Nature* **254**: 245–247

Unger C, Kress S, Buchmann A and Schwarz M (1994) γ-irradiation-induced micronuclei from mouse hepatoma cells accumulate high levels of the tumour suppressor gene p53. *Cancer Res* **54**: 3651–3655

Warenius HM, Britten RA and Peacock JH (1994*a*) The relative cellular radiosensitivity of 30 human in vitro cell lines of different histological type to high LET 62.5 MeV (p->$Be^+$) fast neutrons and 4 MeV photons. *Radiother Oncol* **30**: 83–89

Warenius HM, Browning PGW, Britten RA, Peacock JA and Rapp UR (1994*b*) C-raf-1 proto-oncogene expression relates to radiosensitivity rather than radioresistance. *Eur J Cancer* **30**: 369–375

Warenius HM, Jones MD and Thompson CCM (1996*a*) Exit from $G_2$ phase after 2 Gy gamma irradiation is faster in radiosensitive human cells with high expression of the *RAF*-1 proto-oncogene. *Radiat Res* **146**: 485–493

Warenius HM, Seabra LA and Maw P (1996*b*) Sensitivity to cis-diamminedichloroplatinum in human cancer cells is related to expression of cyclin D1 but not c-Raf-1 protein. *Int J Cancer* **67**: 224–231

Whitaker SJ, Ung YC and McMillan TJ (1995) DNA double strand break induction and rejoining as determinants of human tumour cell radiosensitivity. A pulsed-field gel electrophoresis study. *Int J Radiat Biol* **67**: 7–18

Wurm R, Burnet NG, Duggal N, Yarnold JR and Peacock JH (1995) Cellular radiosensitivity and DNA damage in primary human fibroblasts. *Int J Radiat Oncol Biol Phys* **30**: 625–633

Zhen W, Denault CM, Loviscek K, Walter S, Geng L and Vaughan ATM (1995) The relative radiosensitivity of TK6 and WI-L2-NS lymphoblastoid cells derived from a common source is primarily determined by their p53 mutational status. *Mutat Res* **346**: 85–92

*© Cancer Research Campaign 1998*